# Tag-mediated effect on the dynamics of social influence

**DOI:** 10.1371/journal.pone.0338598

**Published:** 2025-12-12

**Authors:** Xiaochen He, Guanzhao Cheng, Jiali Lu

**Affiliations:** 1 School of Public Policy and Administration of Xi’an Jiaotong University, Xi’an, Shaanxi Province, China; 2 Institutes of Science and Development, Chinese Academy of Sciences, Beijing, China; 3 Public Policy & Management of Tsinghua University, Beijing, China; Tokyo Institute of Technology: Tokyo Kogyo Daigaku, JAPAN

## Abstract

The dynamics of social influence carry significant sociological and economic implications and have been examined across various disciplines. However, the dynamics of social influence within tag-mediated systems remains underexplored. This study proposes a generalized networked urn model that incorporates tagging effects, extending current understandings of self-reinforcing and self-correcting mechanisms in social dynamics. Mathematical derivations show that tag-related parameters—the share of individuals with a given tag, the probability of being tag-driven, and the effect of tag difference—interact with social influence to shape convergence outcomes. Depending on context, tags can either reinforce early advantages or act as a corrective force that drives outcomes toward balance. Simulation results further indicate that tagging effects may weaken path dependence under certain conditions, reducing market inequality and improving predictability, while in other cases they may sustain disparities. These findings underscore the moderating role of tags in collective dynamics, offering theoretical insights into the social influence processes.

## Introduction

Social influence plays an important role in understanding social dynamics, and it has been employed as the main theory in explaining different aspects of human behaviors like crowdfunding [1], jury deliberations [2] or knowledge diffusion [3]. Olson [4] discusses how the free-riding mechanism impedes collective action, suggesting that factors such as social influence, which may seem irrational, can nonetheless drive success when evaluating Marx’s theory. In a list of new studies, the manifestation of social influence can be also found in political pressure, lobbying or partisan divisions [5,6].

The impact of social influence on the collective system (comprising various groups or parties) is often explained by self-reinforcing feedback mechanism [7,8]. For example, individuals tend to purchase a product based on its popularity, and its popularity may become entrenched as more people continue to buy it. Prior studies have demonstrated that when an initial bias exists, social influence can cause inferior goods to persist in the market while superior alternatives fail [9–11]. This phenomenon aligns with the Matthew Effect, whereby initial advantages or disadvantages amplify over time, reinforcing existing disparities [12]. Self-reinforcing mechanisms have applied to explain diverse real-world contexts, such as social conventions [8] and inequality in cultural markets [13]. More recent research, however, suggests that social influence can also involve self-correcting dynamics. When popularity becomes entrenched, individuals’ intrinsic preferences may partially rebalance the market and offset self-reinforcement [1,14]. Such self-correcting processes have been observed in domains including children’s strategic behavior [15] and media crises [16].

These two mechanisms appear contradictory, as certain structural features of social systems—such as thresholds [17,18], negative relationships [14], and limited social information [1]—can constrain self-correction from counteracting reinforcement. A limitation of much of this research is the assumption of individual homogeneity, even though heterogeneity across populations plays a crucial role in social dynamics [19]. “Tags”, also referred to as phenotypic markers, provide a key concept for capturing heterogeneity [20,21]. Becker [22] described tags as socially constructed markers shaped by environmental calibration, which confer positive or negative identities. McPherson and Smith-Lovin [23] further showed that tags influence decision-making through homophily. In game theory, numerous tag-based models have confirmed the “green beard effect,” whereby individuals prefer to cooperate with others sharing similar tags [24–27]. Castillo [28] extended this perspective to social influence, noting that political party affiliation exemplifies a tag-mediated effect. Yet, how tags shape the specific pathways of social influence remains an open question. Addressing this gap could advance social identity theory within information science.

Mathematical models have long been employed to study the dynamic of social influence [29–35]. Among these, the urn model is a widely used framework for explaining the self-reinforcing mechanism under social influence [36]. Pólya [37] first proposed the urn model based on a ball-drawing process, and its variants have been applied to diverse real-world scenarios [38–41]. The present study extends this tradition by proposing a generalized urn model that incorporates individual tags, enabling analysis of tag-mediated effects on both self-reinforcement and self-correction. One application is to explain the dynamic role of ethnocentrism in collective action—a phenomenon insufficiently addressed in social identity theory. More broadly, the model illustrates how social science and computational approaches can be integrated.

Unlike most existing urn models, which assume individuals make choices solely by imitating others, we consider that individuals sometimes act according to intrinsic preferences [42]. Accordingly, each decision maker follows others’ choices with probability *p*,and follows innate preferences with probability 1 – *p*. Building on this premise, we propose a tag-based urn model to examine how tags mediate self-reinforcing and self-correcting dynamics.

The main contribution of this study is twofold: first, to extend models of social influence to hypernetwork structures; and second, to demonstrate how tag-mediated effects shape the emergence of collective dynamics. The remainder of the paper is organized as follows. Section 2 introduces the tag-based urn model. Section 3 presents the theoretical analysis of tag-mediated effects. Section 4 reports simulation results under different parameters. Section 5 concludes with discussion.

## Urn model in tagged networks

### Evolution of urn model

**Fig 1:**
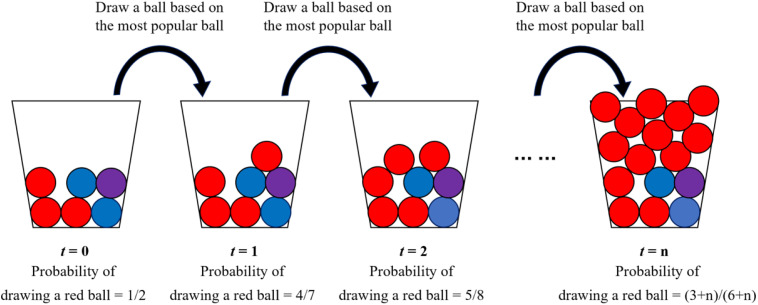
An illustration of the Pólya urn model.

Economic behaviors, social changes, and cultural activities usually involve the social dynamics of social influence [7,13,17,34,43–47]. Under the impact of social influence, self-reinforcement may generate herding effects with non-Pareto bias [48,49]. This leads to path dependence, in which popularity becomes locked in and shapes subsequent market trajectories [7,46]. The Pólya urn model provides a stochastic framework for understanding such path dependence driven by self-reinforcement [50]. In the basic Pólya urn model [37], the entire market or social system is initialized as an urn containing some balls of specific colors. Subsequently, each individual randomly selects a ball from the urn, returns it to the urn, and then places a new ball of the same color into the urn. Fig 1 illustrates the dynamics of this process. The probability of randomly selecting a red ball at **t* *= 0 is 1/2. If a red ball is drawn, then the probability of drawing a red one at *t* = 1 increases to 4/7. Over time, the red color gains increasing popularity, forming a self-reinforcing process. Although the specific color that becomes dominant is unpredictable, Pólya [37] emphasized that the path dependence—where early advantages drive later popularity—is inevitable.

**Fig 2:**
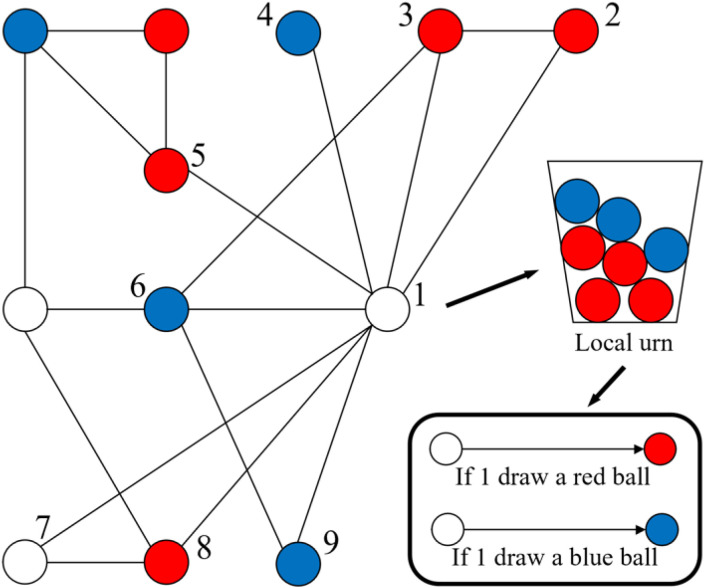
An illustration of the networked Pólya urn model.

Modern economic and social theories require the analysis of social structures with more complex characteristics [51]. The development of network analysis methodologies has expanded the scope of social influence research beyond the traditional confines of cultural transmission. Recently, the urn model has been discussed in the context of structured populations [40,52]. Instead of drawing balls from a single urn, Banerjee et al. [53] proposed a networked version in which each agent maintains a local urn that collects information from its neighbors. This formulation allows for a more detailed understanding of how structural boundaries shape the accessibility and spread of influence. In the context of epidemic spreading, Hayhoe et al. [40] introduced a network urn model where each node randomly draws a ball from its local urn and then selects a ball of the same color as the drawn one. The networked version of the Pólya urn model is depicted in Fig 2.

### Introducing tags in the urn model

**Fig 3:**
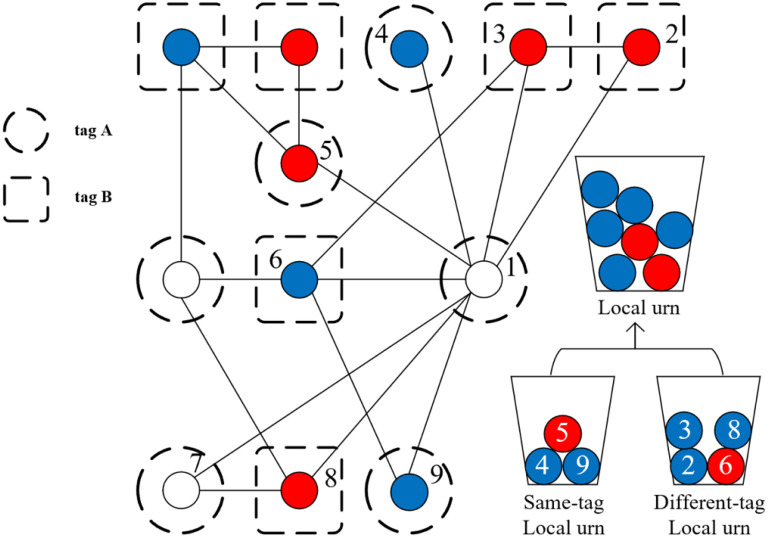
An illustration of Pólya urn model in tagged networks.

Unlike conventional networked processes, the presence of diverse tags generates a broader range of mechanisms. Tags represent the interests, stances, and other characteristics of individuals, and thereby shape social relationships and the diffusion of information [54,55]. The extend of social influence often depends on tag similarity [25], with both homophily and xenophobia playing critical roles in tag-mediated dynamics [23,56]. Xenophobic tendencies can be reinforced by categorization and inference biases, particularly when inductive inferences or stereotypes are applied inappropriately [57]. In societies where tags are highly salient, focusing solely on homophily is insufficient. Tag differences (e.g., race, political affiliation) can also lead to divergent choices, making xenophobia an essential component of social influence models [57,58]. Nevertheless, most prior discussions remain theoretical. To address this gap, we explicitly incorporate xenophobia into our model, with further elaboration provided in the mathematical derivation and simulation sections. In this study, we propose a generalized urn model that integrates tag-mediated effect. Each node in the network is assigned a unique tag, representing various attributes such as different community membership, political party, and organizational affiliation. The tags of neighboring nodes may be either identical or distinct. Accordingly, each node places balls drawn from neighbors with the same tag into its same-tag local urn, while balls from neighbors with different tags are placed into a different-tag local urn. Fig 3 illustrates the tag-based urn process.

To capture the characteristics of the tag-structured network, the parameters of the proposed model are summarized in Table 1. These parameters are introduced progressively during the theoretical analysis. Importantly, individuals neither simply follow the crowd nor mimic others mindlessly like lemmings when making decisions. Instead, they may hold personal preferences that shape their responses to others’ choices. In a social experiment on influence, Salganik et al. [13] distinguished between an independent group—whose members made choices based on personal preferences—and an influenced group—whose members were guided by others’ behavior. The experiment suggests the existence of a coefficient *p* that mediates between the two: with a probability *p*, an individual is influenced by others, while making choices based on its own inclination with a probability of 1-*p*. Intuitively, an influence-dominated environment may lead to a self-reinforcement dynamics, while the personal bias can introduce a self-correcting force. In the present study, the parameter *p* is incorporated into the proposed urn model to balance these dynamics.

**Table 1:** Parameters and their meanings.

Parameter	Meaning
*i*	Index of ball colors
*p*	Probability that an agent is influenced by others
*q*_*i*_	Agent’s intrinsic value preference for color *i*
*n*	Number of agents
${PS}_{i(n)}^{j}$	Probability of selecting color *i* on *j* occasions when there are *n* agents
*δ*	Effect of tag difference
*ζ*	Probability of being tag-driven
*B*	Number of individuals who have made a choice
*M*_*i*_	Market share of color *i*
*N*_*i*_	Number of agents choosing color *i*
*λ*	Proportion of agents with a given tag when two types of tags exist

In the traditional urn model, individuals may add several balls of the same color when making a choice, which eventually leads to the dominance (or solidification) of a particular color through repeated updates and iterative state changes—an effect analogous to disease contagion [13]. In the model presented in this paper, each agent makes a decision only once, allowing us to focus more clearly on the influence of the tag effect on the overall system. Specifically, we employ the variable ζ to represent the probability that an agent is tag-driven. To examine how tag differences influence decisions when individuals rely on others, we also employ a variable *δ* as the effect of tag difference. The value of *δ* ranges from [−1, 2]. When *δ* > 0, different tags exert a moderating effect such that the individual places *δ* balls of the opposite color into the local urn. When *δ* < 0, tag differences do not generate hostility; instead, the individual places -*δ* balls of the same color into the local urn. In the proposed framework, a global urn represents the entire system, while each individual *l* maintains a local urn. For each neighbor *j* of *l* with the same tag as *l*, *j* adds a ba*ll* of its chosen co*l*or to *l*’s local urn. For each neighbor *k* of *l* with a different tag as *l*, the contribution depends on whether *l* is tag-driven. If *l* is tag-driven and δ>0, *k* puts *δ* ba*ll* of the opposite co*l*or; if δ<0, *k* puts -*δ* balls of the same color. If *l* is not tag-driven, *k* follows the same rule as neighbor *j*. Subsequently, with a influence probability *p*, agent *l* selects a ball from its local urn and adds a ba*ll* of the same color to the global urn. Conversely, with probability 1-*p*, the agent instead chooses according to its intrinsic preference and contributes a ball of that preferred color to the global urn.

### Theoretical deduction

**Fig 4:**
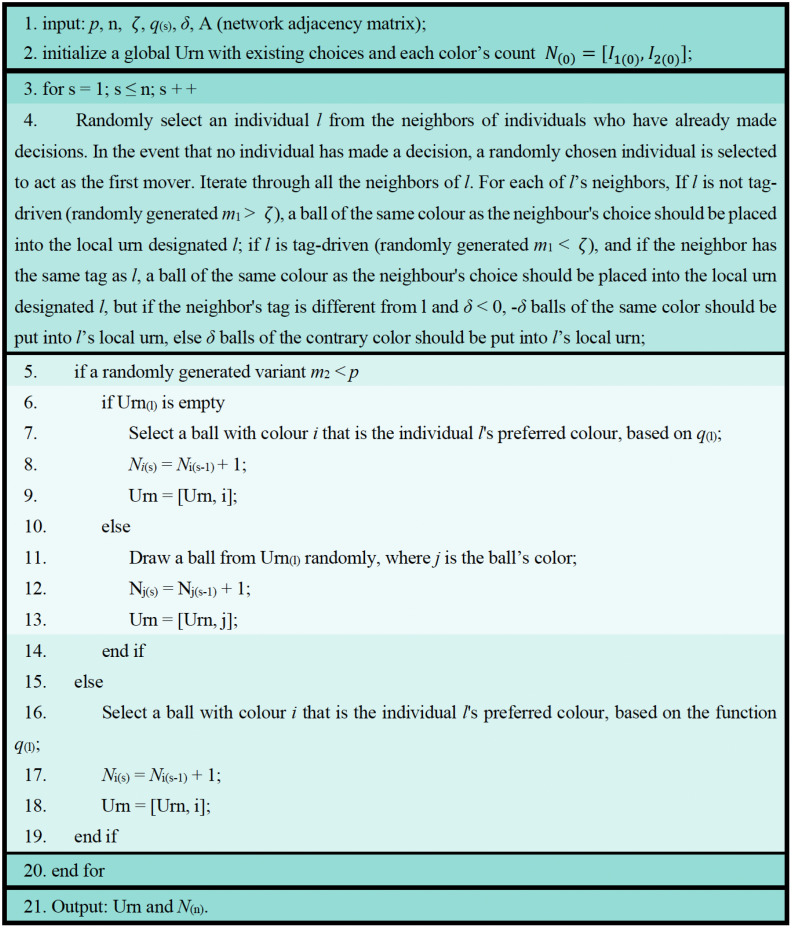
Model algorithm.

To simplify the mathematical derivations in network evolution, the analysis in this subsection is based on a mean – field approximation. This means that we have replaced the difference in degree distribution between nodes with the average connection characteristics of the entire network. Our theoretical analysis assumes a fully connected system, which can be regarded as a special limiting case of the mean-field approximation. To aid comprehension and provide a concise overview of the model, we present a model algorithm diagram as shown in Fig 4.

### Setup and assumptions

To ensure clarity, the model parameters are summarized in Table 1, and their roles are progressively introduced in this section alongside the algorithmic flow in Fig 4. In the context of independent world, for each color *i*, researchers can calculate the intrinsic value preference *q*_*i*_ (corresponding to its market share). If the initial urn is assumed to be empty, the probability that the first mover selects a ball of color *i* is represented by $PS_{i(\text{1})}^{\text{1}}=q_{i}$, while the probability of selecting a ball of a different color is $PS_{i(\text{1})}^{\text{0}}=\text{1}-q_{i}$. We assume that there are two tags and that the probabilities of individuals belonging to either tag are equal (the case of unequal tag probabilities will be discussed at the end of this section). For simplicity, we set the effect of tag difference *δ* = 1, and the probability of an individual being tag-driven is denoted by ζ.

### Recursive probability derivation

In this subsection, we use mathematical derivation to analyze how the probability of individuals choosing a ball with color *i* evolves over time. This provides the basis for calculating the market share of the ball. At the beginning of the hypothetical system, the probability of not choosing color *i* when a new agent joins the system is $PS_{i(\text{2})}^{\text{0}}=PS_{i(\text{1})}^{\text{0}}\cdot \left[p\cdot \left[(\text{1}-\zeta )\cdot \text{1}+\zeta \cdot \frac{\text{1}}{\text{2}}\right]+(\text{1}-p)(\text{1}-q_{i})\right]$, the probability that the color *i* will be chosen once will be $PS_{i(\text{2})}^{\text{1}}=PS_{i(\text{1})}^{\text{0}}\cdot \left[p\cdot \left[(\text{1}-\zeta )\cdot \text{0}+\zeta \cdot \frac{\text{1}}{\text{2}}\right]+(\text{1}-p)q_{i}\right]+PS_{i(\text{1})}^{\text{1}}\cdot \left[p\cdot \left[(\text{1}-\zeta )\cdot \text{0}+\zeta \cdot \frac{\text{1}}{\text{2}}\right]+(\text{1}-p)\cdot \left(\text{1}-q_{i}\right)\right]$, and the probability that the color *i* will be chosen twice will be $PS_{i(\text{2})}^{\text{2}}=PS_{i(\text{1})}^{\text{1}}\cdot \left[p\cdot \left[(\text{1}-\zeta )\cdot \text{1}+\zeta \cdot \frac{\text{1}}{\text{2}}\right]+(\text{1}-p)q_{i}\right]$. The probability of not choosing color *i* when three agents enter the dynamic is $PS_{i(\text{3})}^{\text{0}}=PS_{i(\text{2})}^{\text{0}}\cdot \left[p\cdot \left[(\text{1}-\zeta )\cdot \text{1}+\zeta \cdot \frac{\text{1}}{\text{2}}\right]+(\text{1}-p)(\text{1}-q_{i})\right]$, while that of choosing *i* once is $PS_{i(\text{3})}^{\text{1}}=PS_{i(\text{2})}^{\text{0}}\cdot \left[p\cdot \left[(\text{1}-\zeta )\cdot \text{0}+\zeta \cdot \frac{\text{1}}{\text{2}}\right]+(\text{1}-p)q_{i}\right]+PS_{i(\text{2})}^{\text{1}}\cdot \left[p\cdot \left[(\text{1}-\zeta )\cdot \frac{\text{1}}{\text{2}}+\zeta \cdot \frac{\text{1}}{\text{2}}\right]+(\text{1}-p)\cdot \left(\text{1}-q_{i}\right)\right]$, that of choosing color *i* twice is $PS_{i(\text{3})}^{\text{3}}=PS_{i(\text{2})}^{\text{2}}\cdot \left[p\cdot \left[(\text{1}-\zeta )\cdot \text{1}+\zeta \cdot \frac{\text{1}}{\text{2}}\right]+(\text{1}-p)q_{i}\right]$, and that of choosing color *i* three times is $PS_{i(\text{3})}^{\text{3}}=PS_{i(\text{2})}^{\text{2}}\cdot \left[p\cdot \left[(\text{1}-\zeta )\cdot \text{1}+\zeta \cdot \frac{\text{1}}{\text{2}}\right]+(\text{1}-p)q_{i}\right]$. In a scenario that there are *n* agents (n≥2), the probability of selecting *i* can be expressed as Equation (1), where ${PS}_{i(n)}^{j}$ represents the probability of selecting color *i* on *j* occasions when there are *n* agents.


$$\begin{array}{c} PS_{i(n)}^{j}=PS_{i(n-1)}^{j}\cdot [p\cdot (1-\zeta )\cdot 1+\frac{\text{1}}{\text{2}}\zeta ]+(1-p)(1-q_{i})],ifj=0\\ PS_{i(n)}^{j}=PS_{i(n-1)}^{j}\cdot [p\cdot (1-\zeta )\cdot \frac{n-1-j}{n-1}+\frac{\text{1}}{\text{2}}\zeta ]+(1-p)(1-q_{i})]\\ +PS_{i(n-1)}^{j-1}\cdot [p\cdot (1-\zeta )\cdot \frac{j-1}{n-1}+\frac{\text{1}}{\text{2}}\zeta ]+(1-p)q_{i}],if1\leq j\leq n-1\\ PS_{i(n)}^{j}=PS_{i(n-1)}^{j-1}\cdot [p\cdot (1-\zeta )\cdot 1+\frac{\text{1}}{\text{2}}\zeta ]+(1-p)q_{i}],ifj=n \end{array}$$ (1)

Equation (1) describes the state of a nascent market where no initial choices have been made, and outcomes depend primarily on the decision of the first mover. In contrast, in a mature market with *B* individuals who have already made decisions, suppose that *B_i_* people of them have chosen color *i*. When *n* new agents enter the system, the probability that a total of *j* + *B_i_* individuals select color i across the entire system can be expressed as Equation (2), which parallels the structure of Equation (1):


$$\begin{array}{c} PS_{i(n+B)}^{j+B_{i}}=PS_{i(n-1+B)}^{j+B_{i}}\cdot [p\cdot (1-\zeta )\cdot \frac{n-1+B-B_{i}}{n-1+B}+\frac{\text{1}}{\text{2}}\zeta ]+(1-p)(1-q_{i})],if\;j=0\\ PS_{i(n+B)}^{j+B_{i}}=PS_{i(n-1+B)}^{j+B_{i}}\cdot [p\cdot (1-\zeta )\cdot \frac{n-1+B-(j+B_{i})}{n-1+B}+\frac{\text{1}}{\text{2}}\zeta ]+(1-p)(1-q_{i})]\\ +PS_{i(n-1+B)}^{j-1+B_{i}}\cdot [p\cdot (1-\zeta )\cdot \frac{j+B_{i}-1}{n-1+B}+\frac{\text{1}}{\text{2}}\zeta ]+(1-p)q_{i}],if\;1\leq j\leq n-1\\ PS_{i(n+B)}^{j+B_{i}}=PS_{i(n-1+B)}^{j-1+B_{i}}\cdot [p\cdot (1-\zeta )\cdot \frac{j+B_{i}-1}{n-1+B}+\frac{\text{1}}{\text{2}}\zeta ]+(1-p)q_{i}],if\;j=n \end{array}$$ (2)

### Market share expectations and limit behavior

From Equation (2), it follows that the probability of an individual choosing color *i* can be expressed as a recursive formula. This implies that the probability of any individual choosing a given color cannot be obtained in closed form. In this section, we use this recursive formulation to derive the expected market share of each color and to examine convergence behavior. Equation (2) can be further applied to compute the market share of each color, denoted by $M_{i}=N_{i}/n$, where *N*_i_ represents the number of agents choosing color *i*. The expected value for the market share of color *i* is given by $E[M_{i(n+B)}]=[\sum_{j=\text{0}}^{n}\;\left[PS_{i(n+B)}^{j+B_{i}}\cdot \left(j+B_{i}\right)\right]]/(n+B)$ when there are *n* + *B* agents involved in the dynamic. This formula provides a theoretical estimate at each iteration. To assess the stability and convergence of the dynamic, we apply a transformation to the probability formulation. Given the the known market share of color *i* at time t=n−1, the probability of the next agent selecting color *i* is $E\left[P_{i(n)}\right]=p\cdot [(\text{1}-\zeta )\cdot M_{i(n-\text{1}+B)}+\frac{\text{1}}{\text{2}}\zeta ]+(\text{1}-p)q_{i}$. Thus, the expected market share for color *i* can be expressed as Equation (3):


$$E[M_{i(n+B)}]=\frac{N_{i(n-1+B)}+1}{n+B}\cdot P_{i(n)}+\frac{N_{i(n-1+B)}}{n+B}\cdot (1-P_{i(n)})$$ (3)

However, Equation (3) does not reveal the asymptotic market share as *n* tends to infinity. To address this, Equation (3) can be rewritten as the recursive Equation (4) by applying the tower property of conditional expectation:


$$E[M_{i(n+B)}]=[1-\frac{P(\zeta -1)+1}{n+B}]\cdot M_{i(n-1+B)}+\frac{p\cdot \frac{\text{1}}{\text{2}}\zeta +(1-p)\cdot q_{i}}{n+B}$$ (4)

As *n → ∞*, we obtain the limiting condition: $\underset{n\to \infty }{\text{lim}}E[M_{i(n)}]=E[M_{i(n-\text{1})}]$, implying the convergence of color *i*’s market share. Let$M_{i(n+B)}=M_{i(n-\text{1}+B)}$, we can acquire $\frac{p(\zeta -\text{1})+\text{1}}{n+B}\cdot M_{i(n+B)}=\frac{p\cdot \frac{\text{1}}{\text{2}}\zeta +(\text{1}-p)\cdot q_{i}}{n+B}$. Solving the equilibrium condition yields: when ζ=0 and **p* *= 1, the model reduces to the traditional Pólya urn model, in which the system can converge to all possible values [37]. In other cases, the limit of *M*_*i*(*n*+*B*)_ is given by $\frac{p\cdot \frac{\text{1}}{\text{2}}\zeta +(\text{1}-p)\cdot q_{i}}{p(\zeta -\text{1})+\text{1}}$. In particular, if ζ=0, the system will converge to the *q*_*i*_, that is, the willingness to participate in group behavior based on individual preferences. If ζ=1, $M_{i(n+B)}=(\frac{\text{1}}{\text{2}}-q_{i})\cdot p+q_{i}$, which is linear in p. For $\frac{\text{1}}{\text{2}}<q_{i}\leq \text{1}$, function *M*_*i(n+B)*_ exhibits a linear relationship with a negative slope; for $\text{0}\leq q_{i}<\frac{\text{1}}{\text{2}}$, it increases linearly as *p* grows. In this context, the probability of social influence *p* does not directly align with either self-reinforcement or self-correction mechanisms. Instead, the dynamics balance intrinsic preferences and external influence, producing more moderated outcomes.

Now, consider the case that there are two types of tags among individuals, Suppose the proportion of agents with one tag is λ, and the proportion with the other is 1-λ. Then the probability that two randomly selected agents share the same tag is $P_{same}=\lambda^{\text{2}}+(\text{1}-\lambda {)}^{\text{2}}=\text{2}\lambda^{\text{2}}-\text{2}\lambda +\text{1}$, and the probability that any two people have different tags is $P_{diff}=\text{2}\lambda (\text{1}-\lambda )=-\text{2}\lambda^{\text{2}}+\text{2}\lambda$. Accordingly, the probability of *j* + *B*_*i*_ individuals selecting color *i* in the entire market can be expressed as Equation (5):


$$\begin{array}{c} PS_{i(n+B)}^{j+B_{i}}=PS_{i(n-1+B)}^{j+B_{i}}\cdot [p\cdot [\zeta \cdot (2\lambda^{2}-2\lambda )\cdot \frac{n-1+B-2B_{i}}{n-1+B}+\frac{n-1+B-B_{i}}{n-1+B}]+(1-p)(1-q_{i})],if\;j=0\\ PS_{i(n+B)}^{j+B_{i}}=PS_{i(n-1+B)}^{j+B_{i}}\cdot [p\cdot [\zeta \cdot (2\lambda^{2}-2\lambda )\cdot \frac{n-1+B-2(j+B_{i})}{n-1+B}+\frac{n-1+B-{\left(j+B_{i}\right)}_{i}}{n-1+B}]+(1-p)(1-q_{i})]\\ +PS_{i(n-1+B)}^{j-1+B_{i}}\cdot [p\cdot [\zeta \cdot (2\lambda^{2}-2\lambda )\cdot \frac{-n-1-B+2(j+B_{i})}{n-1+B}+\frac{j+B_{i}-1}{n-1+B}]+(1-p)q_{i}],if\;1\leq j\leq n-1\\ PS_{i(n+B)}^{j+B_{i}}=PS_{i(n-1+B)}^{j-1+B_{i}}\cdot [p\cdot [\zeta \cdot (2\lambda^{2}-2\lambda )\cdot \frac{-n-1-B+2(j+B_{i})}{n-1+B}+\frac{j+B_{i}-1}{n-1+B}]+(1-p)q_{i}],if\;j=n \end{array}$$ (5)

Given the market share of color *i* at time t=n−1, the probability of next agent choosing color *i* is $E\left[P_{i(n)}\right]=p\cdot [\left[\text{1}+\text{2}\zeta \cdot \left(\text{2}\lambda^{\text{2}}-\text{2}\lambda \right)\right]\cdot M_{i(n-\text{1}+B)}-\zeta \cdot (\text{2}\lambda^{\text{2}}-\text{2}\lambda )]+(\text{1}-p)q_{i}$. Consequently, the expected market share value of color *i* can be expressed as Equation (6):


$$E[M_{i(n+B)}]=[1+\frac{p\cdot [1+2\zeta \cdot (2\lambda^{2}-2\lambda )]-1}{n+B}]\cdot M_{i(n-1+B)}+\frac{p\cdot \zeta \cdot (2\lambda^{2}-2\lambda )+(1-p)\cdot q_{i}}{n+B}$$ (6)

When *n* → *∞*, $\underset{n\to \infty }{\text{lim}}M_{i(n+B)}=M_{i(n-\text{1}+B)}$, and we can acquire $\frac{p\cdot \left[\text{1}+\text{2}\zeta \cdot \left(\text{2}\lambda^{\text{2}}-\text{2}\lambda \right)\right]-\text{1}}{n+B})\cdot M_{i(n+B)}=\frac{p\cdot \zeta \cdot \left(\text{2}\lambda^{\text{2}}-\text{2}\lambda \right)-(\text{1}-p)q_{i}}{n+B}$. Then the limiting behavior satisfies: If tags do not play any role (ζ=0 , or λ = 0 or 1) and *p* = 1, *M*_*i*(*n*+*B*)_ will converge to all possible values, aligning with the findings of the classic urn model [37]. In other scenarios, *M*_*i*(*n*+*B*)_ will converge to $\frac{(\text{1}-p)q_{i}-p\cdot \zeta \cdot \left(\text{2}\lambda^{\text{2}}-\text{2}\lambda \right)}{\text{1}-p\cdot \left[\text{1}+\text{2}\zeta \cdot \left(\text{2}\lambda^{\text{2}}-\text{2}\lambda \right)\right]}$, and when **p* *= 0, the system will converge to the *q*_*i*_, reflecting individual intrinsic preferences.

### Interpretation

In the preceding subsections, we introduced the model variables and carried out mathematical derivations. Here, we interpret the convergent values obtained and discuss their sociological implications. From the convergent formula, when **p* *= 1, the market share converges to *M*_*i*(*n*+*B*)_ = 1/2. This result implies that, as long as xenophobia exists—even at a minimal level—a fully influence-driven tagged network guides the market toward equal division between options. When 0 < *p* < 1, the derivative of the convergence value with respect to λ is $\frac{\text{2}p\cdot \zeta \cdot (\text{2}\lambda -\text{1})\cdot \left(\text{1}-\text{2}q_{i}\right)\cdot (p-\text{1}}{{\left[\text{4}p\cdot \zeta \cdot \lambda (\lambda -\text{1})-p+\text{1}\right]}^{\text{2}}}$, and the derivative with respect to ζ is $\frac{\text{2}p\cdot \zeta \cdot (\lambda -\text{1})\cdot \left(\text{1}-\text{2}q_{i}\right)\cdot (p-\text{1}}{{\left[\text{4}p\cdot \zeta \cdot \lambda (\lambda \;-\;\text{1})-p+\text{1}\right]}^{\text{2}}}$. When $\text{0}\leq q_{i}<\frac{\text{1}}{\text{2}}$, the expected market share *M*_*i*(*n*+*B*)_ will grow as ζ increases. With respect to λ, *M*_*i*(*n*+*B*)_ will grow as $\lambda \in \left(\text{0},\frac{\text{1}}{\text{2}}\right)$ and decrease as $\lambda \in \left(\frac{\text{1}}{\text{2}},\text{1}\right)$. This trend mirrors *P*_*diff*_, implying that when the two tags are more evenly distributed (i.e., higher probability of encountering someone with a different tag), the market share *M*_*i*(*n*+*B*)_ of color *i* increases. When λ = 0 or 1, *M*_*i*(*n*+*B*)_ converges to *q*_*i*_, thus, $M_{i(n+B)}\in \left(q_{i},\frac{(\text{1}-p)q_{i}+\frac{\text{1}}{\text{2}}p\cdot \zeta }{\text{1}-p+p\cdot \zeta }\right)$, and the maximum value of *M*_*i*(*n*+*B*)_ will increase and approach 1/2 as ζ approaches 1 and *q*_*i*_ approaches 1/2. When $\frac{\text{1}}{\text{2}}<q_{i}\leq \text{1}$, *M*_*i*(*n*+*B*)_ will decrease as ζ increases. With respect to λ, *M*_*i*(*n*+*B*)_ will decrease as $\lambda \in \left(\text{0},\frac{\text{1}}{\text{2}}\right)$ and increase as $\lambda \in \left(\frac{\text{1}}{\text{2}},\text{1}\right)$. This trend follows *P*_*same*_, implying that when the tag distribution is less balanced, the market share of color *i* declines. When λ = 0 or 1, *M*_*i*(*n*+*B*)_ converges to *q*_*i*_, thus, $M_{i(n+B)}\in \left(\frac{(\text{1}-p)q_{i}+\frac{\text{1}}{\text{2}}p\cdot \zeta }{\text{1}-p+p\cdot \zeta },q_{i}\right)$, and the minimum value of *M*_*i*(*n*+*B*)_ will decrease and approach 1/2 when ζ approaches 1 and *q*_*i*_ approaches 1/2. When *q*_*i*_ = 1/2, the convergence value of *M*_*i*(*n*+*B*)_ is equivalent to 1/2. Finally, taking the extremum of *M*_*i*(*n*+*B*)_ with respect to *p* yields $\frac{\left(\frac{\text{1}}{\text{2}}-q_{i}\right)\cdot \zeta }{(\text{1}-p+p\cdot \zeta {)}^{\text{2}}}$, indicating that the extremum of *M*_*i*(*n*+*B*)_ will approach 1/2 as *p* increases.

**Fig 5:**
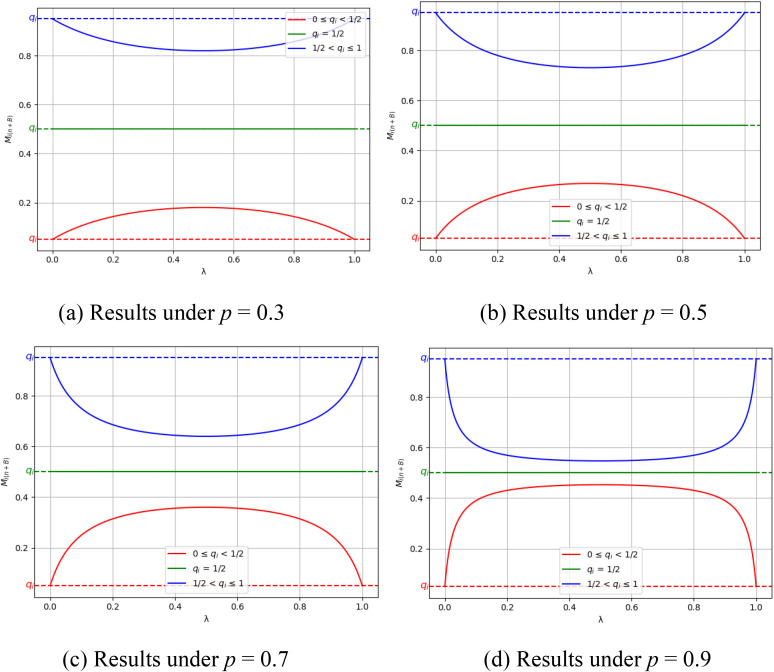
Convergence values of market share. Panels (a)–(d) show results under different probabilities of social influence: (a) *p* = 0.3, (b) *p* = 0.5, (c) *p* = 0.7, and (d) *p* = 0.9.

Fig 5 illustrates these convergence trends. The results show that when tags are homogenized, market shares reflect individual preferences. In contrast, differentiated tags allow such biases to be diluted. The parameter ζ intensifies this effect by modeling xenophobia: as ζ increases, more individuals adopt xenophobic decision rules. The parameter *p* representing susceptibility to influence, amplifies this tendency and accelerates convergence toward equal shares. From a sociological perspective, these findings suggest that in tagged networks, xenophobia can weaken market bias when intrinsic preferences *q*_*i*_ would otherwise favor inferior products. In this sense, xenophobic tendencies within tagged networks can counteract individual bias. However, when *q*_*i*_ represents the intrinsic value of a product, xenophobia risks skewing the market toward inferior outcomes.

### Experiments

The theoretical deduction presented in Section 3 focused on the convergence of dynamics given the optimal initial state and infinite time. In this section, we conducted a series of simulations that illustrate collective dynamics under finite time and various initial conditions, thereby mimicking more realistic markets. The simulations were executed in Python on a computer with a 1.20-GHz CPU and 16.00 GB of RAM, with each parameter iterated 1,000 times.

We generate an Erdős-Rényi random network comprising 1,000 nodes with a density of *ρ *= 0.05 [59]. Tags “A” and “B” were assigned equally, such that 500 nodes carried tag A and 500 carried tag B. To examine the effect of initial bias, the initial urn contained 10 balls under three conditions: (1) a “correct start” with 9 red balls and 1 blue ball, (2) an “equal start” with 5 red and 5 blue balls, and (3) a “false start” with 1 red and 9 blue balls, in which the inferior product initially dominates. Ten nodes were randomly chosen as initial movers. For each initial condition, we ran ten trials with different first-mover sets, and each trial was repeated 100 times with different influence paths. To represent product quality differences, red and blue balls were assigned values *q*_*1 *_= 1 and *q*_*2 *_= 0, respectively.

**Fig 6:**
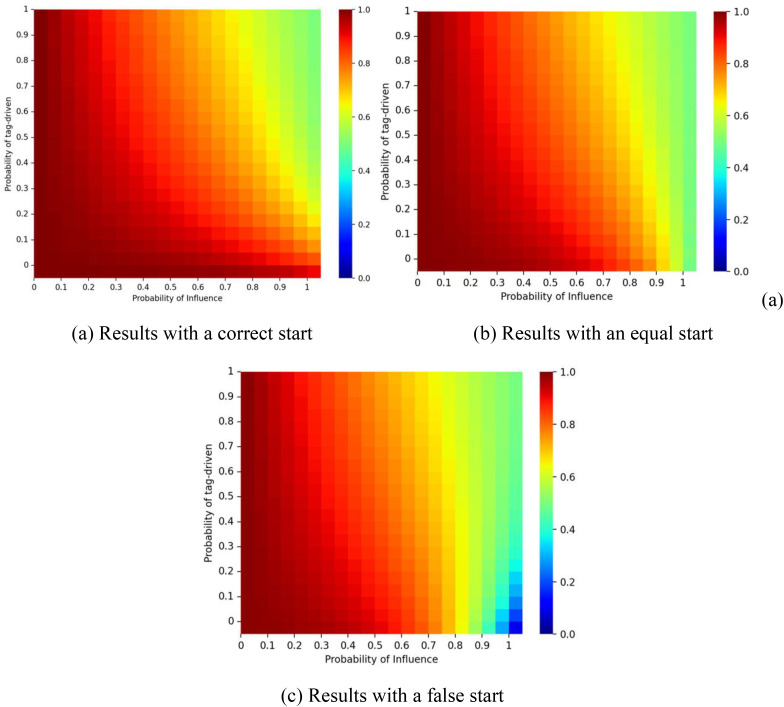
Proportion of the red balls for different values of social influence probability *p* and tag-driven probability ζ.. Panel (a) shows results for a correct start (initial urn: 9 red, 1 blue); panel (b) for an equal start (5 red, 5 blue); and panel (c) for a false start (1 red, 9 blue). The proportion of individuals with tag A is fixed at λ = 0.5, and the effect of tag difference is δ = 1.

Fig 6 illustrates the interaction of the probability of social influence *p* and tag-driven probability ζ on the dynamics. When *p* is small, the market share of red balls remains close to *q*_*1 *_= 1. As *p* increases and ζ remains small, the market share of the red balls gradually approaches the initial start. Because simulations were capped at 1,000 iterations, convergence differs from the theoretical case with infinite time. When ζ is small, most nodes reduce the tagging effect and follow homophilic logic. In this context, higher *p* creates conditions for mimicry, strengthening self-reinforcement and amplifying early decisions (e.g., “red → red → red”). By contrast, as the value of ζ increases, the proportion of red balls approaches 0.5. ζ thus reflects the extent to which xenophobia moderates homophily. At higher ζ, larger *p* values may stimulate opposition to early choices, disrupting path dependence and producing more balanced diffusion patterns (e.g., “red → blue → red”). This outcome is consistent with Equation (6).

**Fig 7:**
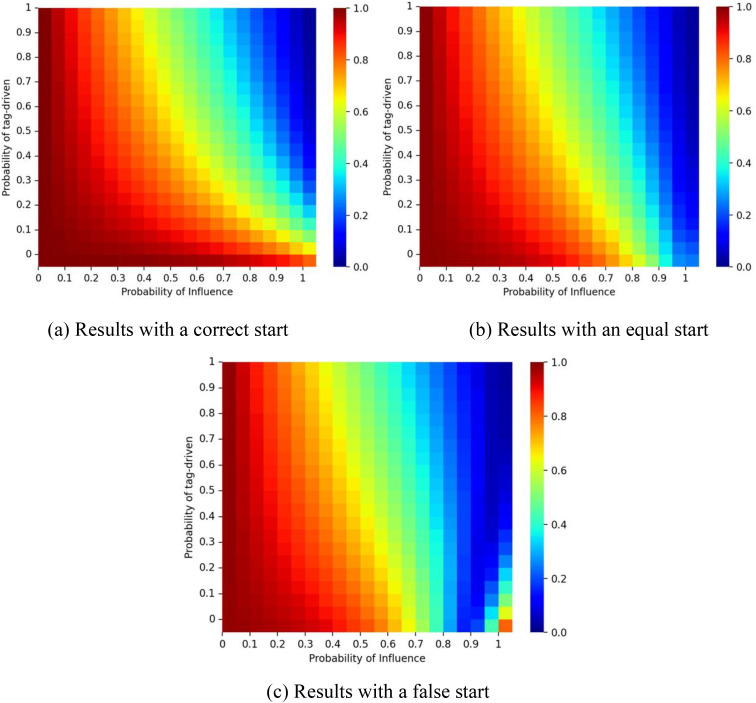
Gini coefficient for different values of *p* and ζ. Panel (a) shows results for a correct start (initial urn: 9 red, 1 blue); panel (b) for an equal start (5 red, 5 blue); and panel (c) for a false start (1 red, 9 blue). The proportion of individuals with tag A is fixed at λ = 0.5, and the effect of tag difference is δ = 1.

To further assess the effect of social influence on market inequality within the tagged network, fig 7 illustrates the interplay between *p* and ζ on the Gini coefficients of the market shares. The Gini coefficients can be formulated as $G_{p,\zeta }=\sum_{i=\text{1}}^{ini}\;\sum_{j=\text{1}}^{exp}\;\left|\frac{m_{red}-m_{blue}}{m_{red}+m_{blue}}\right|/(ini\cdot exp)$, where *m*_*red*_ represents the number of red balls, *m*_*blue*_ represents the number of blue balls, *ini* represents the number of initialization states per experiment and *exp* represents the number of experiments. Results show that as ζ increases, market shares approach 50%. However, because the red ball represents the higher-quality product (**q₁* *= 1), this equal division may be considered inefficient. In such cases, tagging effects can enable inferior products to retain market share. In such cases, tagging effects can enable inferior products to retain market share. Conversely, when the initial condition favors the inferior product and **p* *> 0.9, larger ζ values help the superior product regain dominance, supporting a self-correcting mechanism.

**Fig 8:**
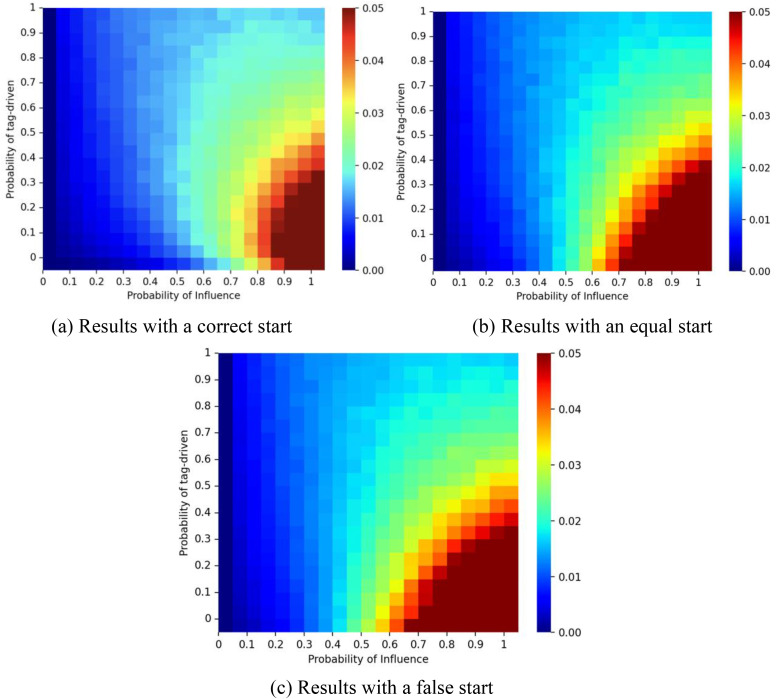
Unpredictability for different values of *p* and ζ. Panel (a) shows results for a correct start (initial urn: 9 red, 1 blue); panel (b) for an equal start (5 red, 5 blue); and panel (c) for a false start (1 red, 9 blue). The proportion of individuals with tag A is fixed at λ = 0.5, and the effect of tag difference is δ = 1.

Path dependence is a key concern in studies of social influence [13]. To examine this, we ran multiple trials under different influence paths. Path dependence was measured by unpredictability across trials, defined for each color *s* as $u_{s,r}=\sum_{i=\text{1}}^{W}\;\sum_{j=i+\text{1}}^{W}\;\left|M_{s,i,r}-M_{s,j,r}\right|/\left(\begin{array}{c} W\\ \text{2} \end{array}\right)$, where *M*_*s,i,r*_ denotes the share of color *s* in trial *i* under initial condition *r*, and *W* = 100 represents the total number of trials. The average unpredictability is then $u_{s}=\sum_{r}^{R}\;u_{s,r}/R$, where **R* *= 10 is the number of initialization scenarios. The overall unpredictability is then calculated as $U=\sum u_{s}/\text{2}$ across the two colors [14]. Fig 8 illustrates the unpredictability results corresponding to Fig 6. When **p* *= 0, the outcome of the system is fully predictable and path-independent. As *p* increases and ζ decreases, unpredictability rises, suggesting stronger path dependence. This reflects fragmentation into many small-scale groups where local imitation dominates, producing instability. When ζ exceeds a threshold, tagging consolidates individuals into a few larger groups, reducing path dependence and improving predictability. However, poor initial conditions, strong social influence, and weak tagging power together increase the risk of unpredictable market dynamics.

**Fig 9:**
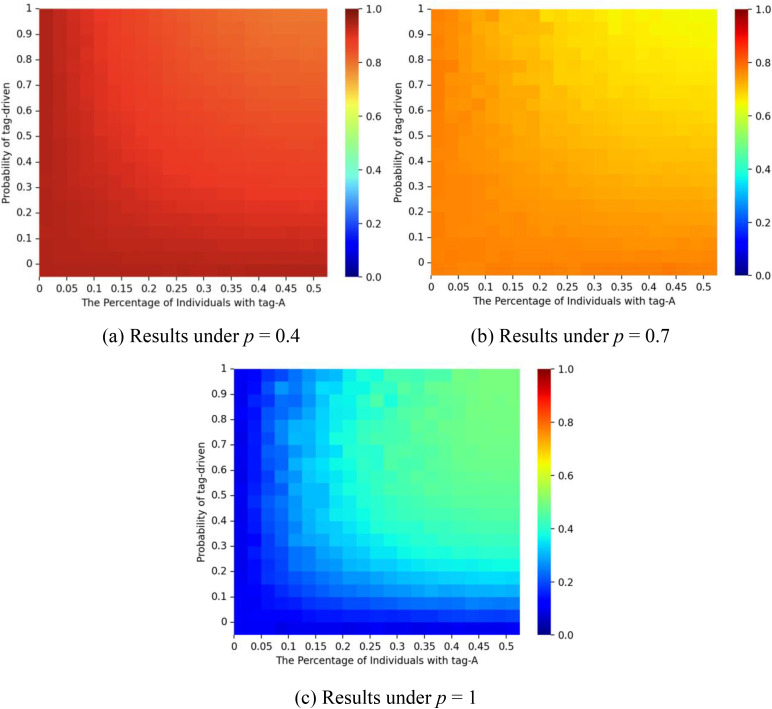
Proportion of the red balls for different values of λ and ζ. Panel (a) shows results when the probability of social influence is p = 0.4; panel (b) when p = 0.7; and panel (c) when p = 1. The effect of tag difference is fixed at δ = 1. The system is initialized with a false start (1 red ball and 9 blue balls).

We further explored how the proportion of tags in the tagged network affects the dynamic. Fig 9 shows the crossover effect of the percentage of individuals with tag-A λ and tag-driven probability ζ at varying *p*. We find that λ and ζ tend to make the red ball market share converge to 0.5. When *p* is very high, a dominant tag can allow inferior products to prevail, while balanced tag proportions limit this risk. When *p* is smaller, a dominant tag can help the superior product maintain a larger share.

**Fig 10:**
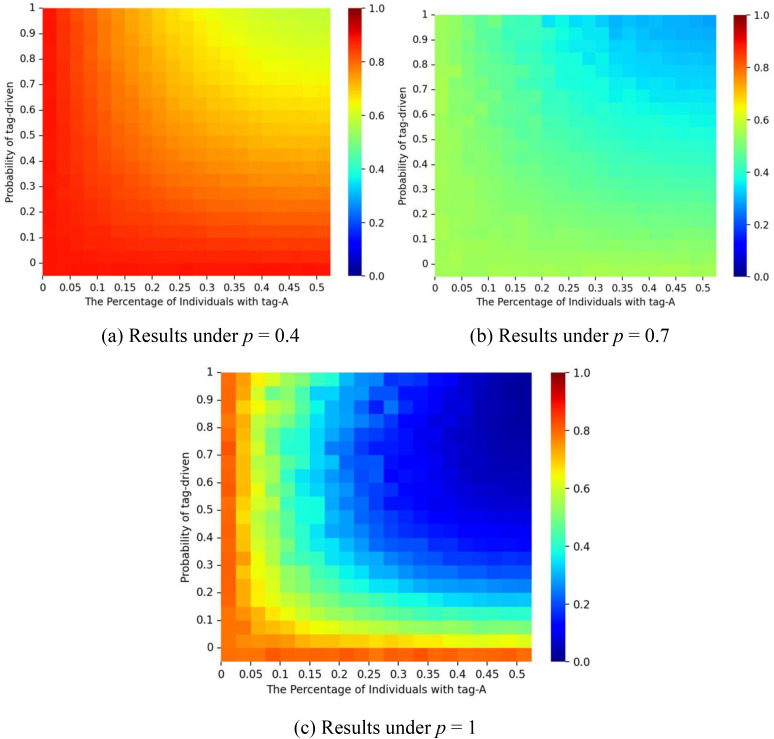
Gini coefficient for different values of λ and $\begin{array}{c} \zeta \end{array}$. Panel (a) shows results when the probability of social influence is p = 0.4; panel (b) when p = 0.7; and panel (c) when p = 1. The effect of tag difference is fixed at δ = 1. The system is initialized with a false start (1 red ball and 9 blue balls).

Fig 10 illustrates the interplay between λ and ζ on Gini coefficients. Larger values of both λ and ζ generally promote self-correction and reduce inequality, while small values sustain self-reinforcement. The dominant mechanism depends on product quality *q*_*i*_.

**Fig 11:**
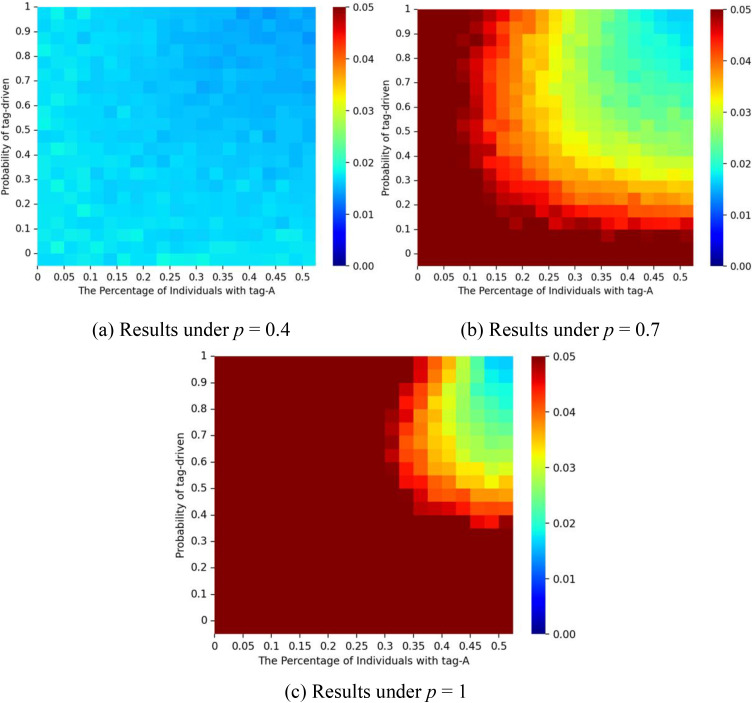
Unpredictability for different values of λ and $\begin{array}{c} \zeta \end{array}$. Panel (a) shows results when the probability of social influence is p = 0.4; panel (b) when p = 0.7; and panel (c) when p = 1. The effect of tag difference is fixed at δ = 1. The system is initialized with a false start (1 red ball and 9 blue balls).

The effects of λ and ζ on unpredictability are shown in Fig 11. When tag sizes are balanced and ζ is large, markets shift toward large-group communities, where xenophobia reduces path dependence. By contrast, high *p* with weak tag effects or homogeneous societies can produce strong path dependence and instability.

**Fig 12:**
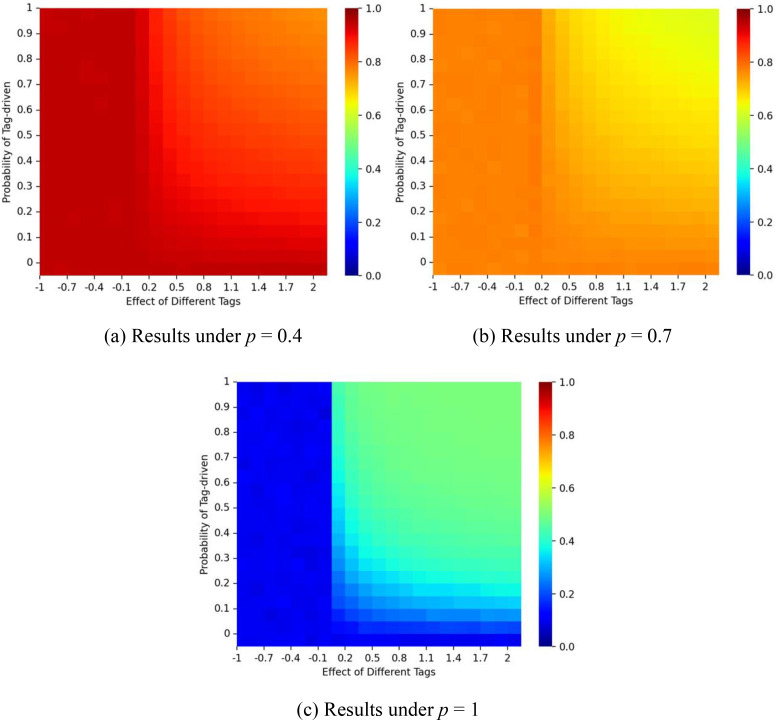
Proportion of the red balls for different values of δ and ζ. Panel (a) shows results when the probability of social influence is p = 0.4; panel (b) when p = 0.7; and panel (c) when p = 1. The proportion of individuals with tag A is fixed at λ = 0.5, and the dynamics are initialized with a false start (1 red ball and 9 blue balls).

Finally, we tested the effect of δ, which modulates the intensity of tag differences. Fig 12 illustrates the crossover effects of δ and ζ on the market share of red balls. The red ball market share changes sharply at δ = 0. When δ < 0, xenophobia is absent, and the system resembles a standard urn model with weak homophily. Since δ operates conditionally on ζ, market shares remain stable when ζ is small. When δ > 0, results align with earlier experiments: stronger tag effects shift outcomes toward balance. This suggests that δ can act as a control parameter for guiding market outcomes toward higher-quality products.

**Fig 13:**
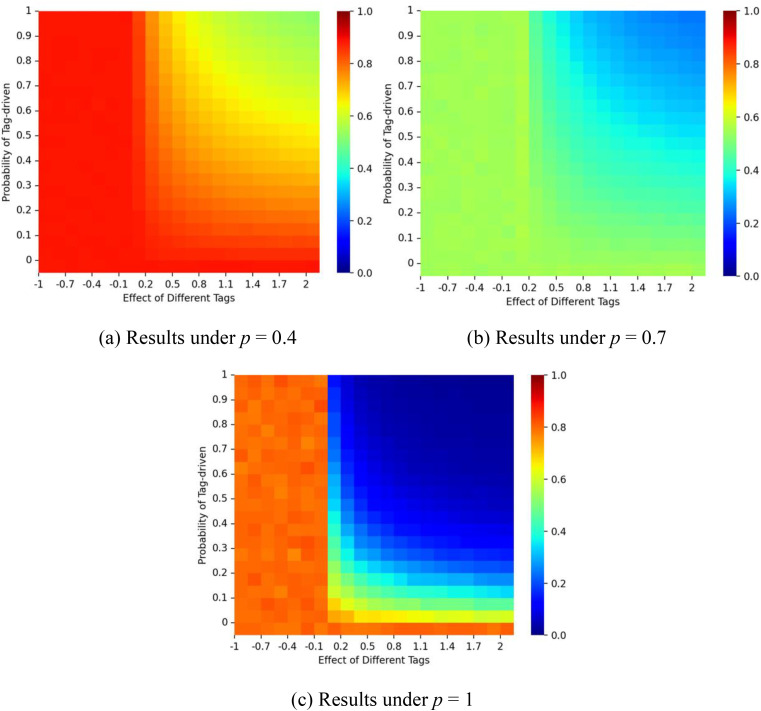
Gini coefficient for different values of δ and ζ. Panel (a) shows results when the probability of social influence is p = 0.4; panel (b) when p = 0.7; and panel (c) when p = 1. The proportion of individuals with tag A is fixed at λ = 0.5, and the dynamics are initialized with a false start (1 red ball and 9 blue balls).

Fig 13 illustrates the effects of δ and ζ on Gini coefficients. When δ < 0, inequality is greater under extreme *p*. When δ > 0, increasing δ and ζ increase reduces inequality. At *p* = 1, shifts around δ = 0 cause large changes in Gini coefficients, indicating that strong social influence amplifies the sensitivity of market equality to tag effects.

**Fig 14:**
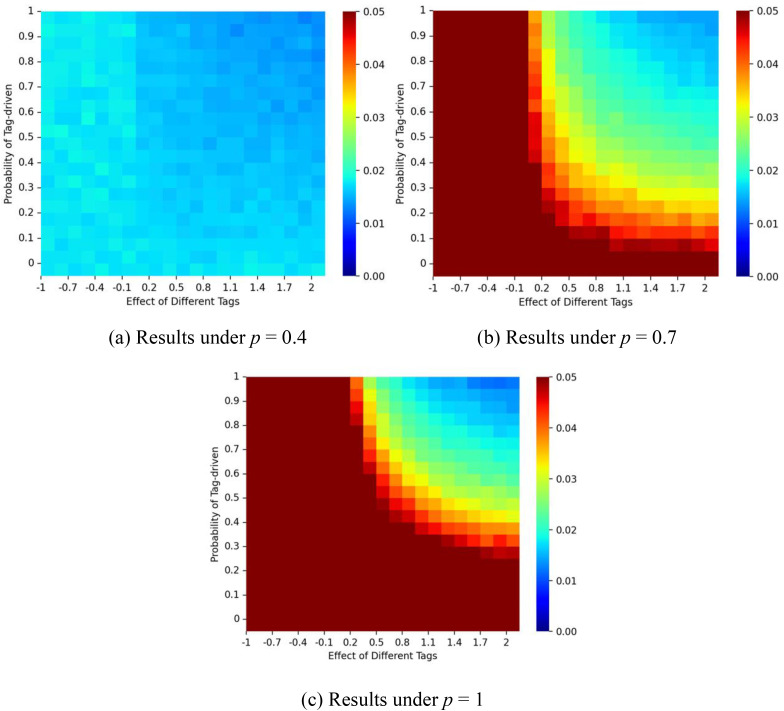
Unpredictability for different values of δ and ζ. Panel (a) shows results when the probability of social influence is p = 0.4; panel (b) when p = 0.7; and panel (c) when p = 1. The proportion of individuals with tag A is fixed at λ = 0.5, and the dynamics are initialized with a false start (1 red ball and 9 blue balls).

Finally, Fig 14 presents a similar outcome to that observed in Fig 11. The combined effect of δ and ζ on unpredictability remains consistent across different *p*. When δ < 0, unpredictability is relatively stable, reflecting homophilic dynamics. Stability can be enhanced either by reducing *p* or by increasing δ and ζ.

## Conclusion and discussion

This study examined the influence of tag-mediated effects on the dynamics of social influence. By discussing the roles of self-reinforcing and self-correcting mechanisms within tagged network, we propose a generalized urn model that incorporates node tags.

Through mathematical derivations, we analyzed system convergence as the number of agents tends to infinity. The results show that given the intrinsic quality of color *i*, the proportion of agents with a particular tag λ, and the probability of being tag-driven ζ, the market share of color *i* converges to $\frac{(\text{1}-p)q_{i}-p\cdot \zeta \cdot \left(\text{2}\lambda^{\text{2}}-\text{2}\lambda \right)}{\text{1}-p\cdot \left[\text{1}+\text{2}\zeta \cdot \left(\text{2}\lambda^{\text{2}}-\text{2}\lambda \right)\right]}$. The analysis further suggests that when tags are homogenized, market shares reflect personal preferences and reinforce existing dynamics. By contrast, when tags shift toward xenophobic attributes (higher ζ), the system tends toward self-correcting mechanisms that balance outcomes more equally.

Simulation experiments, conducted under finite time and artificial initial conditions, were consistent with the theoretical results. The findings highlight the following: (1) the probability of social influence *p* biases markets toward individual preferences; (2) the tag-driven probability ζ promotes more equal outcomes; and (3) the effect of tag difference δ and the tag proportion λ shape the degree and likelihood of xenophobia. Different combinations of these parameters produce distinctive network structures, such as homogeneous small groups, xenophobia-dominated societies, or heterogeneous group systems. Homogeneous systems are more prone to self-reinforcement, while heterogeneous systems exhibit stronger self-correcting tendencies.

Analysis of unpredictability shows that strong social influence increases path dependence and instability, whereas tagging effects enhance predictability. In this way, tagging can regulate market inequality, balance diffusion dynamics, and improve system stability. When network attributes and product qualities together create markets dominated by inferior products or unstable dynamics, targeted interventions may help mitigate risks.

The experiments in this paper are carried out iteratively based on the Erdős-Rényi random network, which indicates that the experimental results may vary when the network structure is different. For example, in the scale – free network model, the central nodes, serving as information hubs in the network, are highly likely to have the same label as their “followers” [60,61]. This implies that the selection of central nodes has a substantial influence on the convergence of market share. Due to the large degree variance in the scale – free network, and the fact that networks with a large variance have lower robustness against attacks (such as biases in the selection of central nodes), the unpredictability of the system may increase [62]. In the small – world network, as the rate of social influence spreading accelerates and the number of global shortcuts increases, it may lead to a increase in the system’s unpredictability [63]. In future work, we plan to extend our analysis to various network topologies to gain a deeper understanding of how structural properties influence the dynamics of the tag effect on social influence.

The results also provide a formalized tool for studying social influence in tagging systems and extend the explanatory scope of Tajfel’s [64] social identity theory. Social identity theory posits that individuals categorize into groups, identify with their in-group, and develop in-group preferences and out-group biases. Our model reflects these dynamics by showing how tagging shapes decision-making: social influence propagates within groups but is constrained across groups, reflecting the interplay of in-group preference and out-group bias.

Several limitations should be noted. First, our analysis is theoretical and simulation-based. Future work should extend the study of tag-mediated influence to signed networks, which could help explain structural balance in evolutionary dynamics. Second, empirical validation is needed: carefully designed social experiments that incorporate appropriate network attributes and tags would allow testing of the model’s predictions under real-world conditions.

## Supporting information

S1 File Code of simulation experiments. (ZIP)
